# A Non-puerperal Uterine Inversion Due to an Endometrial Polyp

**DOI:** 10.7759/cureus.72353

**Published:** 2024-10-25

**Authors:** Marta Almeida, Paula Bettencourt, Sílvia Couto, Mariana Aleixo, Joana Rego

**Affiliations:** 1 Obstetrics and Gynecology, Hospital Garcia de Orta, Almada, PRT; 2 Obstetrics and Gynecology, Hospital de Santo Espírito da Ilha Terceira, Angra do Heroísmo, PRT

**Keywords:** haultain technique, huntington technique, non-puerperal uterine inversion, postmenopausal bleeding, uterine polyp

## Abstract

Non-puerperal uterine inversion is a rare complication, and its incidence is not well documented in the current literature. The most common risk factor is the presence of submucosal fibroids. Nonetheless, any endometrial pathology can precipitate this inversion and may also have a malignant etiology. A detailed anamnesis and structured complementary diagnostic exams, along with a surgical team prepared for the resolution of this gynecological condition, are essential. This case presents a 76-year-old woman with non-puerperal uterine inversion following the prolapse of a uterine mass. Treatment occurred in two phases: firstly, by vaginal approach with excision of the uterine mass, which was causing vaginal bleeding, pain, and urinary retention. This was followed by an abdominal hysterectomy after the diagnosis of uterine inversion by MRI and the apparent exclusion of malignant etiology.

## Introduction

Uterine inversion is defined as an invagination of the uterine fundus through the cervix, potentially reaching the vulva [1]. It represents a unique clinical entity with challenging diagnosis and treatment [1]. Uterine inversion can be divided into puerperal (the most common) and non-puerperal or gynecological [2]. The majority of cases are obstetric complications, with gynecological inversion being rare, with no reported estimated incidence in the literature [1]. Uterine inversion is further subdivided into four stages: stage 1, incomplete inversion, where the uterine fundus remains in the uterine cavity and does not pass through the cervix; stage 2, complete inversion, where the uterine fundus has surpassed the cervix; stage 3, total inversion, where the uterine fundus reaches the vaginal introitus; and stage 4, where the uterus and vaginal walls are involved in the inversion, which is considered total [3].

Non-puerperal uterine inversions are often caused by uterine tumors (benign or malignant) [1]. Although uterine fibroids are the most frequently described cause, it is reported in the literature that malignant causes can reach 20%-32% of cases [1,2,4]. Therefore, whenever possible, a histological diagnosis and imaging study should be obtained before surgical treatment [4].

The most common clinical presentation includes abnormal uterine bleeding (AUB), pelvic pain, urinary disorders, and the presence of a palpable mass in the vagina, which can occur in many other gynecological conditions [4]. However, the presentation varies, and patients may present with signs of infection or life-threatening uterine bleeding [4].

The primary diagnostic imaging modality is gynecological ultrasound, but magnetic resonance imaging (MRI) is the most sensitive examination for the diagnosis of uterine inversion [4].

The definitive treatment for non-puerperal uterine inversion is surgical [5]. The surgical technique used varies depending on the surgeon's expertise, with abdominal, vaginal, and combined approaches described in the literature [3,4].

In this case report, we describe a case of non-puerperal uterine inversion, stage 3, in a postmenopausal woman caused by a large polyp, initially managed by excision of the tumor mass using an endoloop technique, followed by total abdominal hysterectomy and bilateral salpingo-oophorectomy.

## Case presentation

A 76-year-old woman was referred to the gynecology emergency department due to the protrusion of a 6-cm mass through the vagina, resulting in urinary retention, pelvic pain, and vaginal bleeding. The patient had been experiencing postmenopausal bleeding for two months but did not seek medical attention. Her medical history included well-controlled hypertension and dyslipidemia, with no prior history of abdominal surgery. Regarding her gynecological and obstetric history, she had undergone four uncomplicated vaginal deliveries.

**Figure 1:**
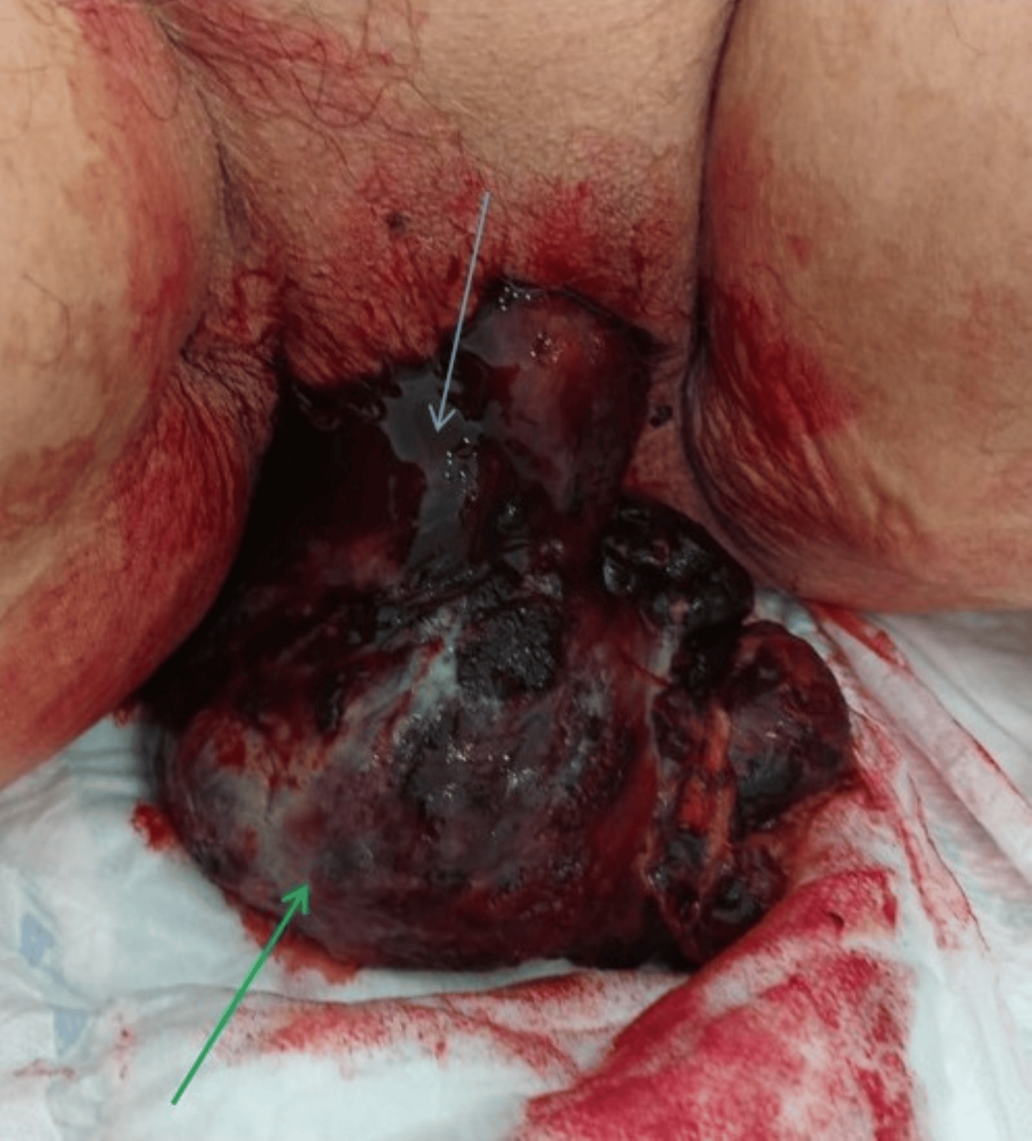
Uterine mass protruding through the vagina (green arrow). The blue arrow points to blood clots. The uterus itself is not visible in the image.

Upon examination, she was hemodynamically stable, with a blood pressure of 136/74 mmHg, a heart rate of 69 beats per minute, and a tympanic temperature of 36.4°C. During the gynecological examination, a 6-cm bloody mass with a necrotic appearance and foul odor was observed protruding from the vagina (Figure 1). A bimanual examination was not feasible. Blood tests revealed a hemoglobin level of 11.4 g/dL and elevated inflammatory parameters, including a white blood cell count of 15600/uL and a C-reactive protein level of 16.5 mg/dL (Table 1).

**Table 1:** Blood test results.

Blood Test	Result	Reference Value
Hemoglobin	11.4 g/dL	12.0-15.5 g/dL
Leukocytes	15.60×10^3^/µL	4.0-11.5×10^3^/µL
Neutrophils	79.9%	50-70%
Lymphocytes	12.9%	22-40%
Platelets	285×10^3^/µL	150-400×10³/µL
Creatinine	0.86 mg/dL	0.6-1.1 mg/dL
Serum electrolytes	-	-
Sodium	134 mmol/L	135-145 mmol/L
Potassium	4.09 mmol/L	3.5-5.1 mmol/L
C-reactive protein	16.5 mg/dL	<0.5 mg/dL

**Figure 2:**
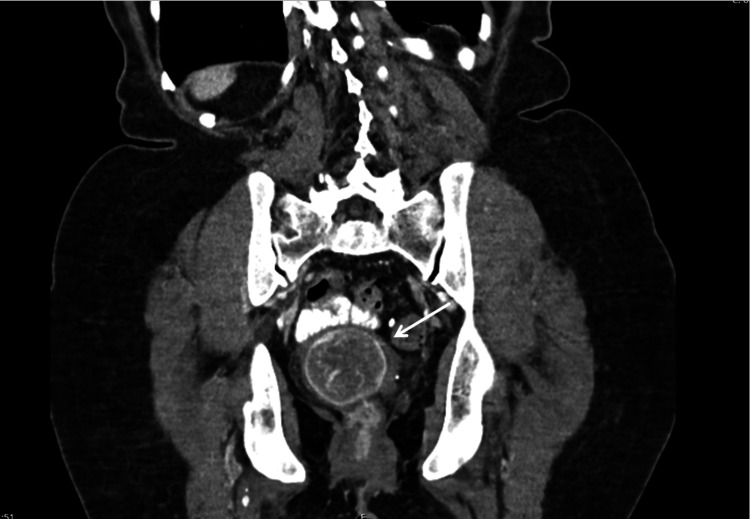
Abdominopelvic computed tomography. The uterus is indicated by the white arrow.

Abdominopelvic computed tomography revealed a well-circumscribed, moderately enhancing endovaginal nodule, possibly a fibroid, measuring 47x42x51 mm, continuous with a non-enhancing heterogeneous mass in the lower vaginal area, protruding. There were no lymphadenopathies or pelvic fluid collections (Figure 2).

**Figure 3:**
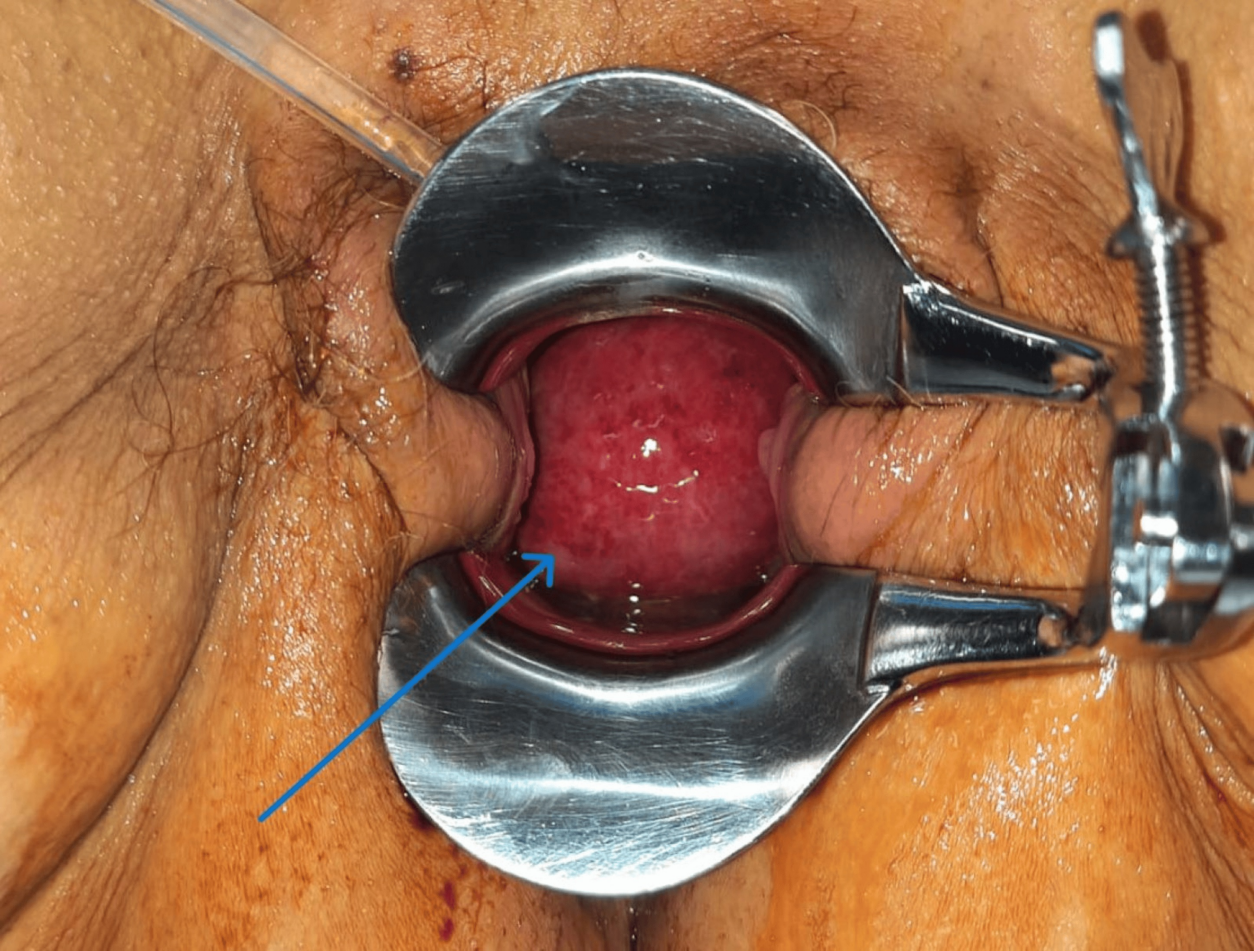
Uterine inversion observed during the speculum examination, following the endoloop excision of the uterine mass. The blue arrow points to the fundus of the endometrium protruding into the vagina.

The patient initiated empiric antibiotic therapy with ceftriaxone 2 g and underwent surgical removal of the uterine tumor for symptom relief and histological diagnosis. During the procedure, the pedicle was ligated with an endoloop and Vicryl® 0 (Ethicon, Bridgewater, New Jersey), and the mass was removed, followed by a reduction of uterine prolapse. After the surgical procedure, a speculum examination allowed visualization of a pink, smooth-surfaced mass protruding into the vagina (Figure 3). The histological diagnosis of the excised mass was consistent with an endometrial polyp.

After the procedure, the patient remained stable with minimal blood loss and experienced two episodes of pelvic mass protrusion, which were manually reduced. On bimanual examination, a firm, protruding mass was palpated, surrounded by a rigid ring, obstructing access to the cervix.

**Figure 4:**
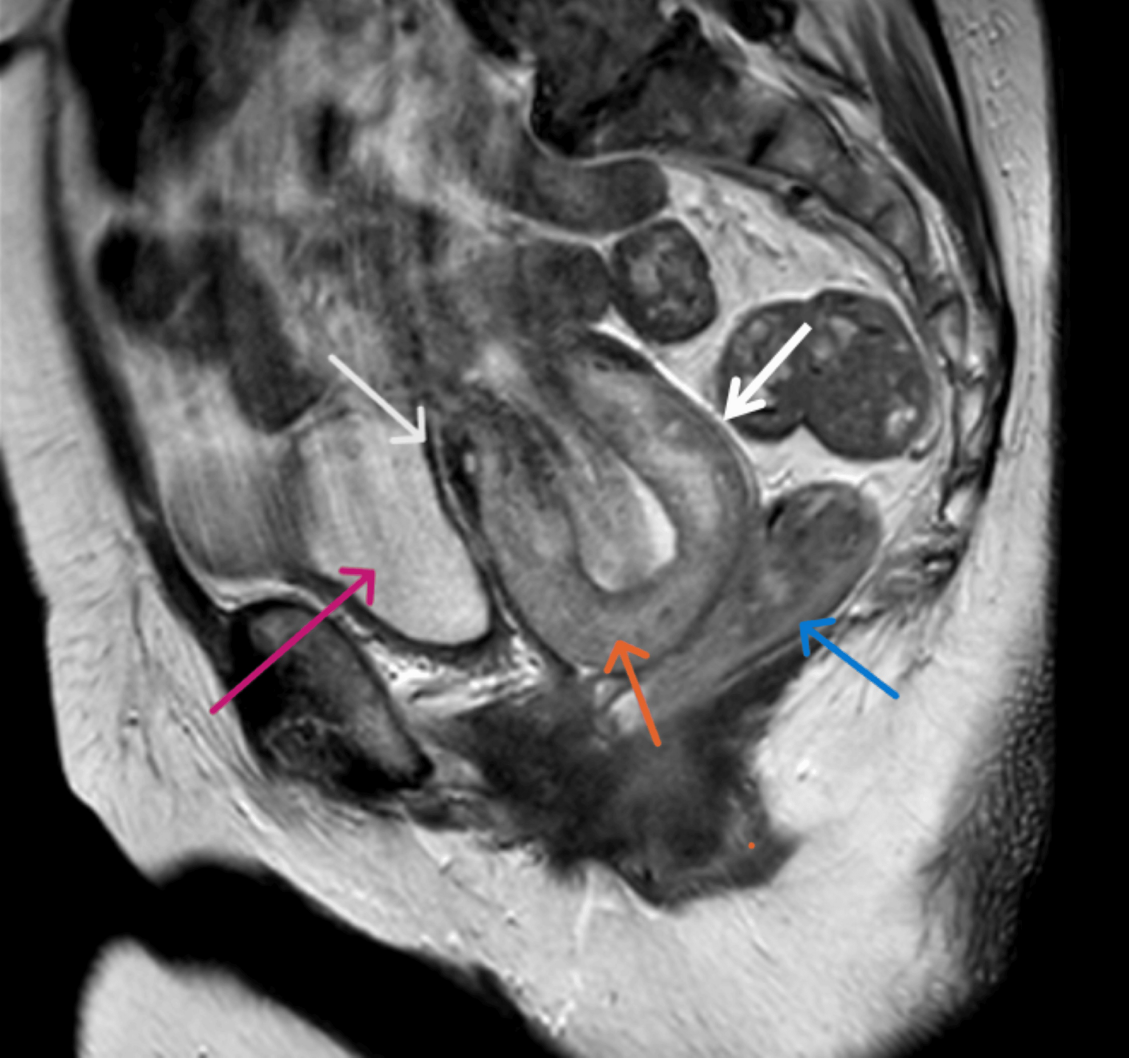
MRI characteristics of uterine inversion in T2-sagittal view. The white arrows point to the anterior and posterior vaginal walls, distended by the uterine corpus; the orange arrow points to the uterine fundus; the pink arrow points to the bladder; the blue arrow points to the rectum.

To further clarify the etiology of the clinical situation, a pelvic MRI was requested (Figure 4), revealing the presence of uterine inversion, with the uterine fundus situated in the lower third of the vagina. The uterus partially occupied the vaginal canal, with the anterior and posterior walls appearing intact. No significant alterations in the myometrium or presence of uterine masses were detected, and there was no evidence of expansive processes. No fibroids, adnexal masses, peritoneal fluid, or lymphadenopathies were identified.

**Figure 5:**
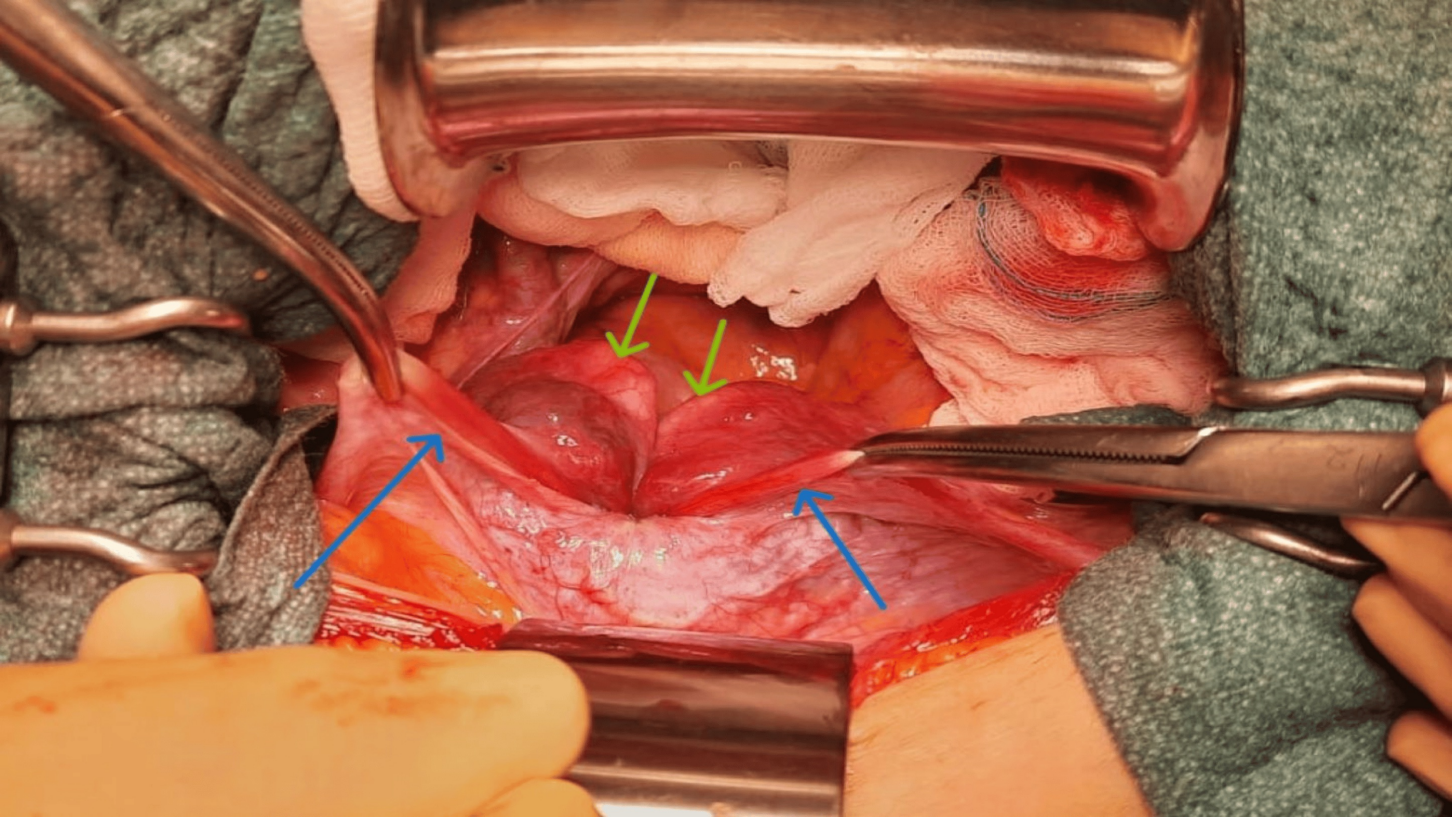
The intraoperative appearance during laparotomy shows Kelly forceps holding the round ligaments, as indicated by the blue arrows. The adnexa were not visible as they were pulled into the interior of the uterine inversion, indicated by the green arrows.

The patient promptly underwent surgery for non-puerperal uterine inversion via laparotomy, considering the limited vaginal access observed during gynecological examination. During surgery, we observed significant distortion of anatomical landmarks, with a complete uterine inversion (grade 2). The adnexa were not visible as they were pulled into the interior of the uterine inversion (Figure 5). Initially, the adnexa were freed from the center of the uterine inversion, and attempts were made to reposition the uterus to its anatomical position by traction on the round ligaments, according to the Huntington technique. However, we were unsuccessful due to a tight cervical ring.

**Figure 6:**
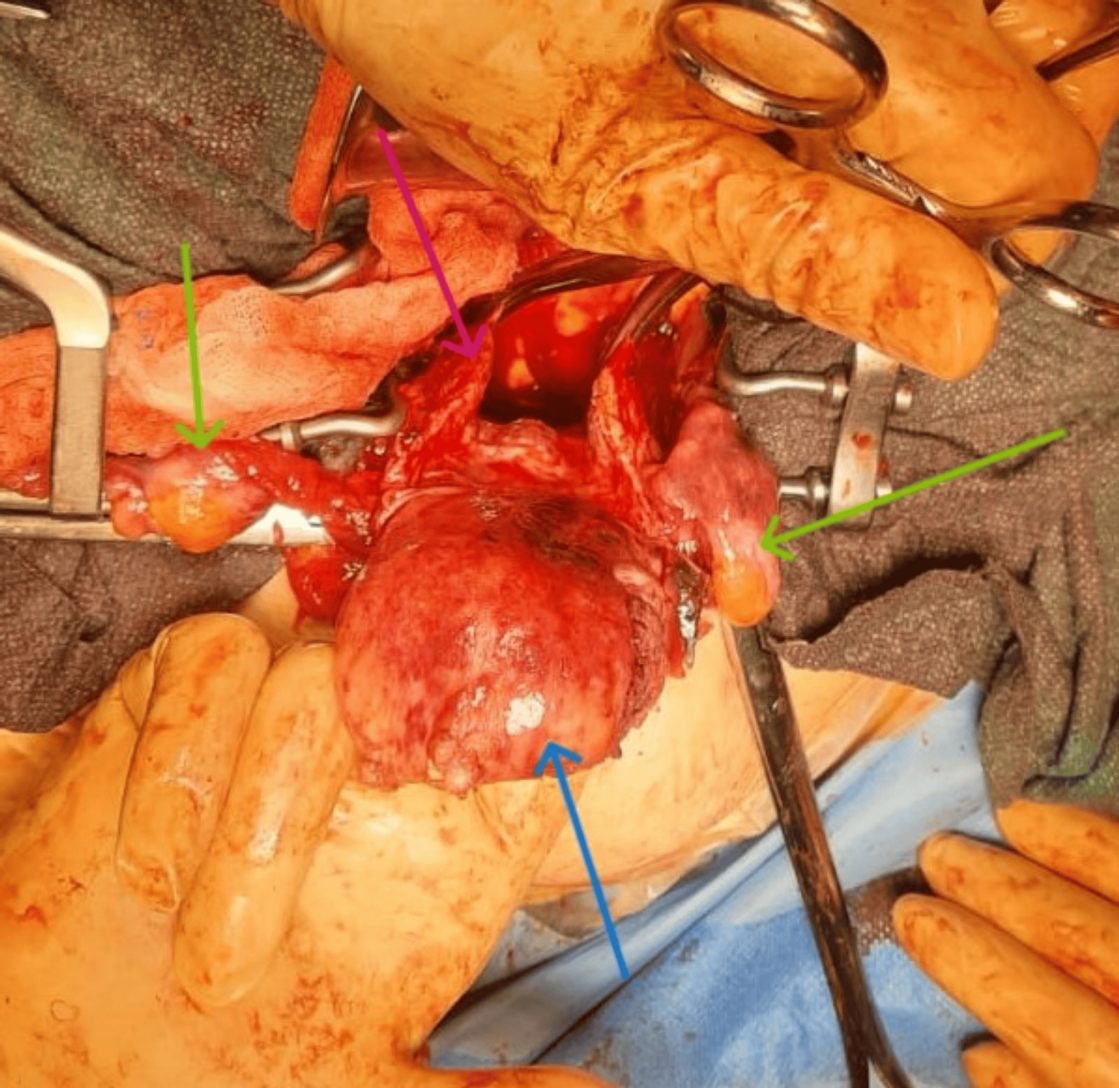
Intraoperative view during laparotomy. A vertical incision was made in the posterior wall of the cervical ring and vagina (pink arrow), following the Haultain technique, facilitating the restoration of normal anatomy. The uterine fundus is indicated by the blue arrow, while the green arrows indicate the adnexa.

Subsequently, after the identification of the ureter's trajectory along the pelvic wall, the infundibulopelvic ligaments were ligated, and the adnexectomy was performed. To allow the uterus's anatomy restoration and hysterectomy, a vertical incision was made in the posterior wall of the cervical ring and vagina, according to the Haultain technique (Figure 6). Subsequently, a total hysterectomy was completed according to standard technique. The patient experienced an estimated blood loss of 250 mL.

Postoperatively, the patient was well and discharged home four days after surgery. At the 6-week follow-up, the patient had fully recovered. The vaginal cuff was well attached, without dehiscence, and fully healed. The abdominal incision had also healed completely. The patient reported no gynecological complaints. The definitive histological analysis of the operative specimen confirmed the absence of malignant pathology in the uterus.

## Discussion

Uterine inversion is a rare but significant clinical condition characterized by the invagination of the uterine fundus through the cervix, potentially extending to the vulva [1]. This case report highlights the complexity and critical aspects of diagnosing and managing this unusual condition.

Non-puerperal uterine inversion is most commonly associated with uterine tumors, both benign and malignant [4]. In our case, the inversion was caused by a large endometrial polyp. While uterine fibroids are frequently cited as the primary cause, malignancies account for a significant percentage (20%-32%) of cases [1,2,4]. This underscores the importance of obtaining a histological diagnosis and conducting thorough imaging studies before surgical intervention [4]. Preoperative MRI and ultrasound are essential diagnostic tools [4]. Ultrasound may show a "Y" or "U"-shaped uterine cavity, but MRI is considered the gold standard due to its superior sensitivity [4]. The MRI confirmed the inversion in this case, revealing a "U"-shaped uterine cavity. These imaging findings were crucial in planning the surgical approach [4].

The definitive treatment is surgical. The cause of the inversion could be managed before or during the same surgical procedure [1,4]. Before hysterectomy, an attempt to reposition the uterus to its anatomical position should be made to facilitate the surgery, but repositioning may be more challenging beyond stage 1 [4,6].

In cases where patients desire fertility preservation, attempts should be made to anatomically reposition the uterus after the removal of the uterine mass that caused the inversion. However, uterine preservation is impossible in approximately half of cases [4].

The most frequently described techniques are Huntington and Haultain for the abdominal approach and Spinelli and Kustner for the vaginal approach [4]. The abdominal approach is the most commonly selected (48.8%-78%) [4,5].

In the case of abdominal techniques, the Huntington technique involves applying traction to the round ligaments and performing digital cervical dilation [2-4]. Suppose a tight cervical ring is encountered or the Huntington technique is unsuccessful, the Haultain technique is recommended, which involves making a vertical incision in the posterior surface of the uterus to bisect the constriction ring to facilitate the repositioning of the uterus to its normal anatomical position [3-6].

For vaginal techniques, Kustner's technique involves opening the posterior cul-de-sac and making a vertical incision at the posterior surface of the cervical ring [3,4]. Pressure is then applied to the posterior uterine surface to achieve anatomical restoration [4]. In contrast, the Spinelli technique requires a vertical incision in the anterior surface at the level of the cervical ring, which is done after opening the anterior cul-de-sac and retracting the bladder to provide adequate access [3-5,7].

Our report describes a non-puerperal uterine inversion in a postmenopausal woman caused by a large polyp, managed in two phases: first, the vaginal excision of the uterine mass using an endoloop technique, followed by total abdominal hysterectomy and bilateral salpingo-oophorectomy. The decision for hysterectomy and adnexectomy was based on the patient's age and our aim to prevent recurrence and ensure that the condition was not malignant. During hospitalization, two episodes of total inversion, stage 3, occurred; however, during surgery, we encountered a complete inversion, stage 2, in which both adnexa were involved. The surgical difficulty arises from the inversion stage and the restoration of normal uterine anatomy.

The abdominal route was selected because, as the uterine fundus invaginates through the cervix into the vagina, it forms a tight cervical ring, making vaginal access difficult. The Huntington and Haultain techniques were employed. Initially, the Huntington technique was attempted, but the Haultain technique was required due to a tight cervical ring. This involved a vertical incision in the posterior uterine surface to facilitate repositioning and subsequent hysterectomy. Successful surgical management underscores the importance of familiarity with multiple techniques to address varying anatomical challenges.

## Conclusions

Uterine inversion is a rare gynecological event that typically results from an endometrial lesion with overgrowth. Diagnosing and treating this condition can be challenging, as it may not be initially identified.

Despite the rarity of these events, this case underscores the importance of considering uterine inversion in the presence of a large vaginal mass protrusion, frequently associated with pelvic pain and AUB. Thorough preoperative imaging and histological diagnosis are crucial for guiding appropriate surgical intervention. It is essential to emphasize the importance of individualized surgical planning and execution in managing such rare gynecological emergencies, aiming for optimal patient outcomes.

## References

[1] Della Corte L, Giampaolino P, Fabozzi A, Di Spiezio Sardo A, Bifulco G (2019). An exceptional uterine inversion in a virgo patient affected by submucosal leiomyoma: case report and review of the literature. J Obstet Gynaecol Res.

[2] Kesrouani A, Cortbaoui E, Khaddage A, Ghossein M, Nemr E (2021). Characteristics and outcome in non-puerperal uterine inversion. Cureus.

[3] Shasindran R, Dharshini N, Aruku N, Sasikala A (2024). Exploring non-puerperal uterine inversion: a case series. Cureus.

[4] Herath RP, Patabendige M, Rashid M, Wijesinghe PS (2020). Nonpuerperal uterine inversion: what the gynaecologists need to know?. Obstet Gynecol Int.

[5] Rosa Silva B, de Oliveira Meller F, Uggioni ML (2018). Non-puerperal uterine inversion: a systematic review. Gynecol Obstet Invest.

[6] Haultain FWN (1908). Abdominal hysterotomy for chronic uterine inversion: a record of three cases. Proc R Soc Med.

[7] Abid S, Dhaou GB, Abdelmoula G (2022). Complete non-puerperal uterine inversion caused by uterine hemangioma: about a case report. Pan Afr Med J.

